# Update on the intriguing roles of AQP4 expression and redistribution in the progression and treatment of glioma

**DOI:** 10.1080/07853890.2024.2401111

**Published:** 2024-09-09

**Authors:** Yu-Long Lan, Shuang Zou, Ruoli Chen

**Affiliations:** a Department of Neurosurgery, Second Affiliated Hospital, School of Medicine, Zhejiang University, Hangzhou, Zhejiang, China; b Key Laboratory of Precise Treatment and Clinical Translational Research of Neurological Diseases, Hangzhou, Zhejiang, China; c Clinical Research Center for Neurological Diseases, Hangzhou, Zhejiang, China; d Key Laboratory of Neuropharmacology and Translational Medicine, School of Pharmaceutical Science, Zhejiang Chinese Medical University, Hangzhou, China; e School of Pharmacy and Bioengineering, Keele University, Staffordshire, UK

**Keywords:** Aquaporin 4, glioma, redistribution, treatment

## Abstract

Aquaporin 4 (AQP4) is abundant in the human brain and has an important role in brain homeostasis and diseases. AQP4 expression has been found to be associated with glioma malignancies. However, the complete understanding of the biological processes and curative importance of AQP4 in glioma remains unclear. The impact of AQP4 subcellular mislocalization on glioma progression and the precise mechanisms regarding AQP4 translocation in glioma need further investigation. In this review, we update recent findings about disturbed AQP4 expression in glioma and explore targeting AQP4 to modulate the glioma progression. Thereafter we discuss some possible mechanisms of action of AQP4 translocations in glioma. The present article offers an appropriate introduction to the potential involvement of AQP4 in the emergence and progression of glioma. Both comprehensive research into the mechanisms and systematically intervention studies focusing on AQP4 are essential. By embracing this strategy, we can obtain a new and insightful outlook on managing cancerous glioma. Although the observations summarized in this review should be confirmed with more studies, we believe that they could provide critical information for the design of more focused research that will allow for systematic and definitive evaluation of the role of AQP4 in glioma treatments.

## 1. Introduction

Glioblastoma multiforme (GBM), the deadliest type of cancer of the brain, carries a bleak prognosis even after receiving conventional treatments such as radiotherapy and chemotherapy. The overall survival rate for patients diagnosed with GBM varies between 12–18 months [1, 2]. Even though numerous studies have found promising biomarkers for GBM, the progress of their use in clinical settings is controvercial due to marker heterogeneity [3]. Further understanding of the mechanism of high malignancy of GBM will help to identify new therapeutic targets for improving the prognosis of GBM patients.

GBM is frequently linked to peritumoral brain edoema (PTBE), which can result in elevated intracranial pressure and a negative prognosis [4]. However, the exact cause of PTBE related to GBM remains unresolved. Numerous mediators, including c-Myc, COX2, nitric oxide, vascular endothelial growth factor (VEGF), aquaporins (AQP) and glucose transporter 1 (GLUT1), were reported to be elevated in PTBE [4, 5]. Water, glycerol and other tiny solutes are conducted by AQP, which are intrinsic proteins of membranes. Mammals have been found to possess 13 AQPs, which are labelled as AQP0 through AQP12. AQP1 and AQP4 are the two primary AQP molecules in CNS and regulate brain water and cerebrospinal fluid (CSF) movement and contribute to cytotoxic and vasogenic edoema [6]. Expression of AQP4 is extremely polarized on the abluminal end astrocytic endfeet that encircle brain blood vessels, in addition to on the subependymal, subpial and basolateral membranes of cells in the ependymal region [7]. Quantitative transcriptome analyses showed that AQP 1, 4 and 9 levels of transcripts are higher in human GBM tumour biopsy specimens than in comparable tissues [8]. This corresponds with their putative functions in glioma survival, cell motility and proliferation [8]. Behnam et al. demonstrated that tumour cell migration, proliferation and angiogenesis were associated with AQP4 [9]. Wang et al. conducted single-cell RNA transcriptome sequencing on 53,059 cells derived from 13 specimens of cancerous glioma and revealed significant heterogeneity among malignant gliomas with various levels of AQP4 expressions [10]. Patients who excessively expressed AQP4 exhibited a low overall survival rate and a poor response to chemotherapy [10]. However, Behling et al. discovered that AQP4 does not affect the general survival rate of IDH-wildtype GBMs [11]. Further attempts should be made to better understand AQP4 activity in the pathogenesis, detection and management of brain tumours, further attempts should be made in this direction.

Our earlier research has shown that AQP4 has a major impact on the prognosis of GBM patients and the effectiveness of anti-glioma drug treatment [12]. In one of our previous investigations, we conducted a systematic analysis that described the key function of AQP4 in the malignant development of glioma. We also highlighted its importance in studying anti-tumour resistance to medications [13]. In glioma, AQP4 protein expression is increased, and its inhibition can significantly inhibit the malignant proliferation of glioma [14]. Recently conducted research has demonstrated that temozolomide can hinder malignant glioma progression by suppressing AQP4 expressions, and suggests this could potentially lead to the discovery of novel therapeutic strategies for GBM [15]. Although AQP4 contributes significantly to developing GBM into a malignant state and to medication resistance [16], further investigation into its molecular mechanisms remains required. Our earlier research demonstrated that targeting AQP4 has a great deal of potential for treating GBM, and it was the first study based on our knowledge to show that AQP4 restriction can considerably increase the responsiveness of GBM treatment with medications [12, 17]. AQP4 regulation can be developed into a new therapeutic approach and be an area of study for treating GBM.

Nevertheless, our research team is still looking for extremely particular AQP4 modulators because none have yet been discovered. Targeting AQP4 subcellular translocation to the cell surface is another approach that can be potentially used in glioma treatment. Further in-depth study of AQP4 modulation is required to elucidate the effective GBM treatment.

## 2. Modulation of glioma progression by AQP4

The AQP4 protein is commonly observed with a distinct morphological structure called an orthogonal array of particles (OAPs), which accumulate within the tetrameric unit [18]. There are two main isoforms of the AQP4 protein, which differ in the beginning codon for the amino acid methionine (M). M23 is the name of the shorter, more prevalent version and M1 is the longer, less dominant form [19]. AQP4 isoforms have been shown to play a role in GBM biology in past investigations [20]. Studies have revealed that OAPs are either missing or disintegrating in GBM. Fallier-Becker et al. demonstrated a negative relationship between the occurrence of OAPs and the severity of malignancy [21]. Simone et al. found that M1-AQP4 played a crucial role in facilitating the invasive capabilities of glioma cells, while the buildup of M23-AQP4 in OAPs was detrimental leading to apoptosis [22]. This data is intriguing because it shows that the increased invasiveness is caused by an increase in matrix metalloproteinase-9 (MMP-9) activity [22], which has previously been connected to glioma cell proliferation and the survival rate of patients [23] and this would be discussed further in detailed in the following sections. In addition, it is worth noting that the AQP4 protein has various isoforms, including the extended M1ex and M23ex canonical M1 and M23. These isoforms impact the protein’s expression, performance and assemblage in OAPs [24]. The mechanism of translational read-through is accountable for producing extended isoforms in the central nervous system (CNS) of humans. Palazzo et al. conducted a study on AQP4ex-KO mice and discovered that AQP4ex could be critical for the attachment of the AQP4 protein to the perivascular astrocytic end-foot membrane domains [25]. The M23 and M1 canonical isoforms, which are present in high quantities in the AQP4ex mouse, form large OAPs. However, these OAPs are not properly localized and are restricted to the astrocytic processes in contact with the neuropile of the brain. It can be assumed that AQP4ex plays a role in the advancing downregulation and improper localization of AQP4 that is noticed in GBM [18].

The mechanism of malignant glioma invasion is complicated and might be regulated by various genes and pathways of signalling throughout multiple stages. The characteristics of tumour cells include decreased adhesion to surrounding cells, heightened accessibility of tumour cells and breakdown of the matrix surrounding the cells [26]. Several key studies have provided results that summarize the impact of AQP4 on encouraging glioma migration and invasion of cells [27–29]. First, glioma invasion and migration depend on cells generating and retracting cell membrane protrusions throughout the outer margin [30]. Interestingly, AQP4 exhibits polarization towards the lamellipodia, which subsequently leads to a rise in the volume and/or number of lamellipodia in cells during the migration process. This phenomenon occurs in areas with swift water movement across the cell membrane [31]. This statement aligns with observations suggesting that ion and transporters’ channels may significantly impact the migration of cells by the polarization of the leading edge of cells in motion. The ions’ movement could create a gradient of osmosis that facilitates the influx of water when cells move [32]. Secondly, AQP4 may play a role in organizing the cytoskeleton. According to a recent research report, there may be a correlation between AQP4 deficiency and actin depolymerization, along with a significant alteration in morphology in both rodent and human cells. [33].

In addition, it has been observed that AQP4 is associated with α-syntrophin, a component of the dystrophin-dystroglycan complex (DDC). The complex comprises utrophin and dystrophin, which act as link-maker within the β-dystroglycan (β-DG) and actin cytoskeleton [34]. The findings indicated that the AQP4 protein is implicated in modifying the cellular cytoskeleton, which may be a crucial factor in the migration of cells. Cell-cell adhesion, which is frequently weakened in various human malignancies, is critical for determining the polarity of cells. It is a known fact that lessened intercellular bonding is essential for promoting cell invasion, making it a crucial stage in the advancement from a confined primary tumour to metastatic cancer. The cadherin-catenin cell adhesion complex controls normal intercellular adhesion [35].

According to Polakis et al. connexin 43 is crucial in intercellular adhesion incidents that depend on calcium. Additionally, connexin 43 is frequently overexpressed in particular forms of cancers [36]. Nicchia et al. conducted a study on astrocytes derived from AQP4 mutant mice to explore potentially new functions of AQP4 and its association with connexin 43 and suggested a potentially useful link between channels of water and junction gaps in the brain [33]. AQP4 may regulate glioma adhesion by communicating with adhesion-associated proteins, including connexin 43. All in all, in addition to its significance in water transport, AQP4 has possibly significant functions in the migration, invasion and regulation of gliomas.

Traditionally, brain tumours were considered immunologically non-reactive due to the immune-suppressive function of the brain [37]. Nevertheless, recent studies have provided compelling evidence that tumour growth can elicit an adequate immune response [38]. Wang et al. have discovered the potential relationship between AQP4 and immune variables that influence tumour progression [39]. They observed that macrophages related to tumours in the patients with elevated AQP4 group tended to become polarized towards M2 macrophages [39]. Additionally, the investigators looked at changes in cell state. They found that in GBM samples, cell status varies depending on the amount of AQP4 expression, demonstrating significant heterogeneity within malignant gliomas with various AQP4 expression ratios [39]. All these have emphasized the importance of comprehending the AQP4-related immune repertoire of the brain to facilitate the development of more effective therapeutics.

## 3. AQP4 dysregulation in glioma

A specific polarity associated with AQP4/OAP is necessary for the proliferation of astrocytes in the brain. It has been demonstrated that this kind of polarity is essential for establishing and/or sustaining the blood-brain barrier (BBB) [7]. In the context of human glioblastoma, the presence of AQP4 in astrocytes may significantly affect the aberrant functionality of the BBB. Wolburg et al. aimed to examine the expression pattern of OAP in both normal and human glioblastoma tissues [40]. The researchers discovered he dense concentration of OAPs was found in standard astroglial end-feet membranes but was not present in glioma membranes of cells, even when they interact with the basal lamina [40]. As demonstrated by Noell et al. disruption of the proteoglycan agrin can prevent adaption, while the development of OAPs depends on the presence of agrin [41]. According to Rauch et al. the absence of agrin could result in the redistributing of AQP4 and impairing the cell’s ability to pass water in the right pathway [42]. This could potentially damage the BBB and result in edoema in the brain [42]. In a different investigation, Noell et al. confirmed that the DDC is essential for appropriately expressing AQP4 at the BBB [43]. Additionally, they discovered that agrin is necessary for AQP4 to be distributed polarized in astrocytes [43].

Studies have demonstrated that cells in glioma exhibit significant AQP4 staining throughout. Nevertheless, the density of OAPs in these cells does not seem as great as in the standard end-foot membrane, even though they are close to vessels [13]. It can be presumed that AQP4 may have independent functions in glioma cells, apart from its role in OAPs. According to reports, it has been observed that AQP4 can separate from OAPs and disperse itself evenly throughout the whole outermost layer of cancer cells [34, 41–43]. More research is required to completely comprehend the practical differences between freestanding AQP4 and AQP4 within OAPs. Furman et al. looked at the properties of membranes that had been freeze-fractured from cells with either the M1 or M1 and M23 isoforms of AQP4 or a combination of both [44]. It is worth noting that the development of OAPs was only observed when both isoforms were transfected. The fascinating finding of AQP4 protein up-regulated combined with OAPs down-regulated may explain the M1 isoform up-regulation in gliomas.

## 4. Potential mechanisms that underlie the redistribution of AQP4 inside cells of glioma

The mislocalization of AQP4 in gliomas is evident [13]. However, the underlying processes are largely unknown. Knowing how AQP4 is controlled may help understand how it is disrupted in GBM. The redistribution of AQP4 sub-cellularly in gliomas may be greatly influenced by the modulation of AQP4 isoform expressions. Different membrane dynamics displayed by each AQP4 isoform could assist in understanding how AQP4 gets dysregulated in gliomas. A single AQP4 molecule was located using quantum dots, and tracking of AQP4 protein has been found to demonstrate easy diffusion of the M1 isoform. The isoform M23, however, remained stable [45, 46].

**Figure 1.**
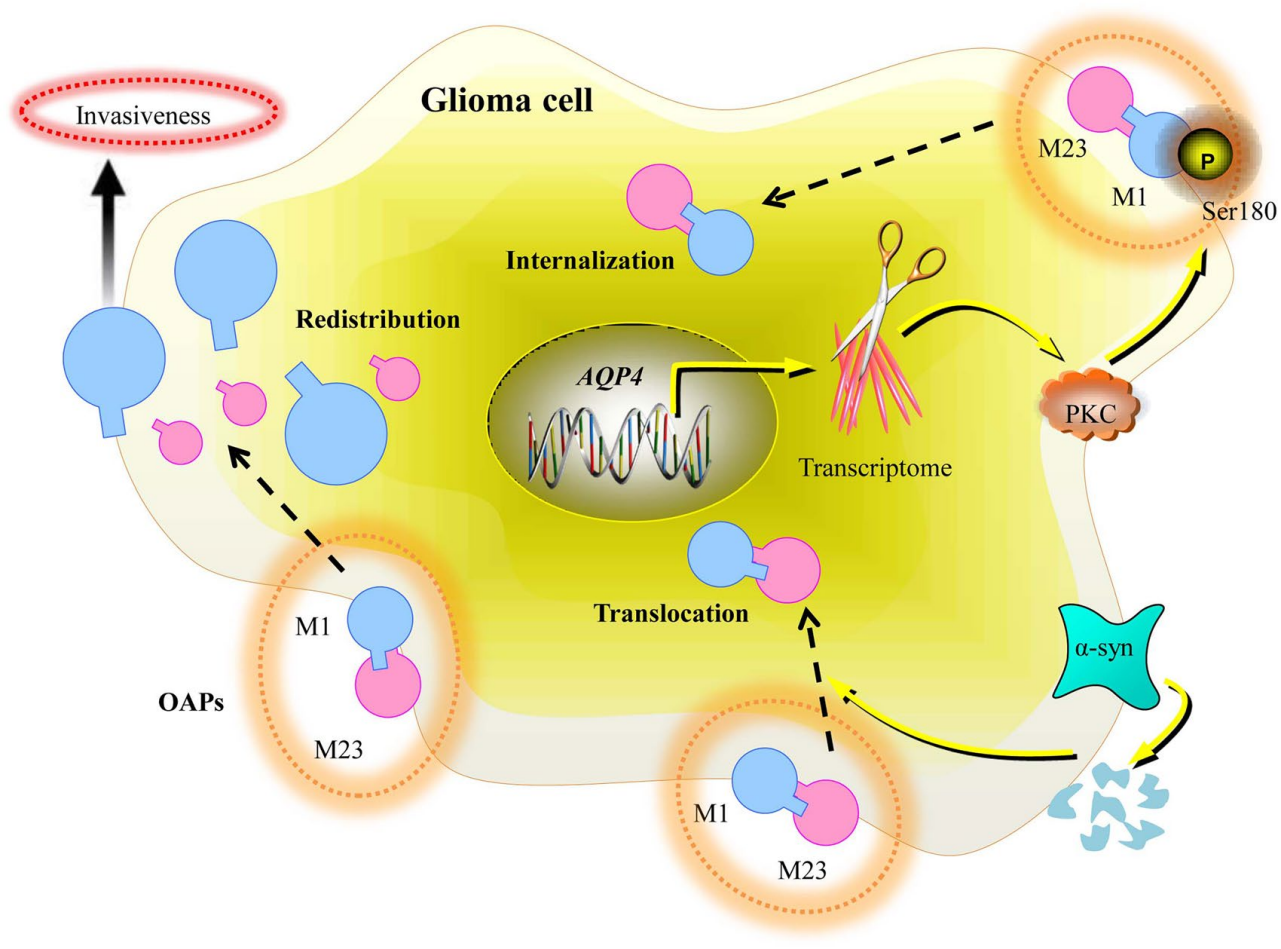
Illustrates the schematic representation of AQP4 subcellular mislocalization. Under the glioma condition, the redistribution of AQP4 and a higher concentration of M1-AQP4 contribute to the intrusion of glioma cells, whereas altered AQP4-M23 may also be deleterious and promote apoptosis. Besides, α-syn anchoring of AQP4 could be another mechanism for regulating AQP4 translocation. Furthermore, post-translational modification of AQP4 could also lead to its subcellular redistribution and internalization. AQP4 Ser180 phosphorylation by PKC is believed to be responsible for this outcome.

Furthermore, the M1-AQP4 and M23-AQP4 aggregating features, may play distinct functional activities and are necessary for AQP4 localization. M1-AQP4 singular tetramers might be required for lamellipodial elongation due to their capacity to permeate across the plasma membrane [45]. Ion transport and water influx, which AQP4 mediates, help cells migrate [47]. M23-AQP4 primarily polarizes AQP4 at astrocyte endfeet because it can generate stable OAPs [45]. Similar molecular events might also exist under glioma (Figure 1). With various pathological circumstances, AQP4-M23 may change due to its organization, surface dynamics and unique localization.

Furthermore, α-syntrophin (α-syn) anchoring of AQP4 could be another mechanism for regulating AQP4 subcellular redistribution. The loss of polarity in AQP4 in tumours may be connected to the loss of the protein that maintains it in place. Researchers have determined the presence of the adaptor protein -syn is linked to an increase in AQP4 around blood vessels [48, 49]. OAPs must be bound to the membrane’s cytoplasmic end to prevent M23-AQP4 diffusion. It was discovered that the PSD95-Discs large-ZO1 (PDZ) binding domain of AQP4 forms connections with other molecules [46]. Investigations have shown that although the overall levels of AQP4 were normal in mice without α-syn, there were substantial decreases in perivascular AQP4 [49, 50]. Additionally, it was shown that these mice’s non-endfeet membranes had unexpectedly higher levels of AQP4 [50]. The results suggest that the removal of α-syn leads to the incorrect placement of AQP4 instead of a complete reduction of AQP4. These results indicate that when the anchoring protein α-syn is lost, there is also an impairment of perivascular AQP4 polarization in glioma.

Furthermore, the subcellular redistribution of AQP4 in glioma might be caused by modification after translation. Studies have demonstrated that the phosphorylation process of AQP4 determines its subcellular localization [51, 52]. Initial research has indicated that when the PKC activator phorbol 12-myristate 13-acetate (PMA) interacts with protein AQP4, it boosts the phosphorylation of AQP4. This, in turn, reduces osmotically-induced swelling of cells [53]. PMA treatment significantly reduced brain water levels in rat ischaemia models *via* inhibiting AQP4 expression [54]. It is believed that the reduction in AQP4 levels, associated with a rise in AQP4 internalization, is caused by the phosphorylation of AQP4 Ser180 by PKC. It was thought that the binding of vasopressin to vasopressin 1a receptors (V1aRs) was necessary for the PKC activation-induced internalization of AQP4 [55]. The stimulation of V1aRs has been shown to support the fast outflow of water in the brain by neurological stimulation [56]. These studies suggest that the subcellular redistribution of AQP4 may occur through a mechanism that depends on PKC.

## 5. The association between AQP4 redistribution and MMP-9-related invasiveness in glioma

As mentioned above, M1-AQP4 played a crucial role in mediating the invasive capabilities of glioma cells, and intriguingly the increased invasiveness has been found to be induced by the increase of MMP-9 activity [22], which has been shown to play a critical role in the mechanisms of glioma invasion [57]. Intriguingly, previous findings have suggested differential expression patterns of MMP-9 and AQP4 in different grades of gliomas, and co-analysis of MMP-9 and AQP4 may help to identify tumour type and their progression stages [58]. However, exploring the precise mechanisms regarding the interaction between AQP4 and MMP-9 could be of great significance for revealing the roles of AQP4 redistribution in glioma progression and treatment.

Previous research has indicated that AQP4 polarity and redistribution depends on an intact dystroglycan complex (DG) [59]. The DG, which is composed of an extracellular α-subunit (α-DG) and transmembrane β-subunit (β-DG), is located on the astrocyte endfeet membrane [60]. The structural integrity of β-DG is essential for basement membrane (BM)-astrocyte endfeet contact and polarization of AQP4 [60]. Remarkably, the extracellular N-terminus of β-DG has been recognized as a specific substrate that is proteolytically cleaved by MMP-9 [61]. Matrix metalloproteinases (MMPs) are synthesized by neurons and glia and released to the extracellular space, where they could exert effects in disrupting neuroinflammatory processes and cellmatrix homeostasis [62]. Interstingly, current research has found a novel dual role for MMP-9 inhibition in regulating aquaporin-4 membrane distribution [63]. The authors demonstrated that the MMP-9 inhibition could exert neuroprotection by employing dual protective mechanism, which encompass a reduction in the invasion of exogenous pro-inflammatory factors. And by modulating the polarity of AQP4, MMP-9 inhibition could facilitate the glymphatic clearance of cytokines, as well as the draining of macromolecules and traffic immunological cells from the CNS into cervical lymph nodes, being a candidate curing strategy against glioma [64].

As mentioned above, the involvement of MMP-9-mediated β-DG cleavage in AQP4 redistribution has long been explored, however, other DG components might also produce similar influences on AQP4 polarization. More future research should be done to verify this effect. Furthermore, AQP4 repolarization has been found to affect the levels of antioxidative stress, mitophagy and apoptotic indicators [65], which could all be closely related to the modulating effects conferred by MMP-9. Thus, more effeorts should be directed towards investigating whether AQP4 redistribution impacts MMP-9-related malignancy in glioma patients.

## 6. Potential therapeutic strategies targeting AQP4 expression and translocation in glioma

The probable involvement of AQP4 in human cancer has drawn much more attention in recent years. As a result, AQP4 inhibitors potentially represent an immensely effective and original class of cancer therapeutics. At this time, AQP4 has no authorized targeted inhibitor [39]. Finding particular AQP4 inhibitors may help develop new mechanism-based treatments for glioma. The three major methods now used to diagnose AQP4 inhibitors are the stopped-flow assay, cell swelling assay and cell shrinkage assay [39]. The fast advancement of artificial intelligence and computer-aided drug research will aid the discovery of newer AQP4 inhibitors. Currently, the literature has documented several AQP4 modulators. Although encouraging experimental outcomes, no one has yet been authorized to be employed among humans (Table 1).

**Table 1.** Overview of potential AQP4 modulators for possible use in glioma treatment.

Series	Year	AQP4 modulator	Model	Major discoveries	Problems or limitations
Zhao et al. [84]	2016	Merformin	Rat glioma model	The study indicated that metformin treatment can protect endothelial cell tight junction and prevent damage to the blood brain barrier, as well as reduce AQP4 expression.	Given the complexity of the dynamics of tumor-induced brain edema *in vivo*, future studies will be necessary to explore other putative mechanisms of metformin in regulation of tumor-induced brain edema.
Chen et al. [85]	2017	TMZ	Glioma cell	The authors identified that TMZ might have therapeutic potential for controlling proliferation, invasion of malignant glioma by inhibiting AQP4 expression through activation of p38 signal transduction pathway.	Future more research regarding the screening of specific small-molecular inhibitors of p38-MAPK-AQP4 pathway may provide new insights for the design of novel mechanism-based therapies for glioma.
Yang et al. [80]	2015	Propofol	Glioma patient	Their results suggested that propofol could inhibit the expression of AQP4.	The limitation of their study was that they did not investigate the PKC mechanism in AQP4 inhibition effect of propofol, which needs to be further verified *in vitro* and *in vivo*.
Lan et al. [12]	2020	CS-6	Glioma cell	As the potential inhibitor of AQP4, CS-6 might mediate the inhibition of GBM via regulating the ATP1A3-AQP4 signaling pathway.	The direct interaction between AQP4 and CS-6 has not been explored, which need further research.

Abbreviations: AQP4: Aquaporin 4; CS-6: Gamabufotalin; TMZ: Temozolomide; Propofol: 2,6-dilsopropylphenol.

TGN-020, known as 2-(Nicotinamide)-1,3,4-thiadiazole with a relatively low molecular weight, has been demonstrated recently as an inhibitor of AQP4 [66, 67]. TGN-020 was found to decrease ischaemic cerebral edoema in live subjects [68]. Unfortunately, there is currently no available data regarding the impact of potential inhibitors on the M1 and M23 isoforms of AQP4. Additionally, there is a lack of knowledge concerning the relationship between inhibiting water transportation and inhibiting the migration of tumour cells [69].

Due to its ability to stabilize metabolism with low side effects, gamabufotalin (CS-6), a main bufadienolide of ChanSu, has been employed to treat cancer [70, 71]. According to earlier studies, CS-6 decreased the expression of AQP4 protein in glioma cells [12]. However, whether AQP4 could be the direct target of CS-6 need further research. In addition, CS-6 is toxic to brain cells.

Tetraethylammonium (TEA) was shown to reduce permeability to water in primary mouse astrocyte cultures [72] and to inhibit water permeability of AQP4 in transfected oocytes [73]. Regrettably, TEA has been found to impact various ion carriers and was demonstrated to significantly alter the electrochemical characteristics of the membranes of cells [74].

Due to its slight but essential blockade of AQP4 water channel functioning, bumetanide, a loop diuretic medicine that restricts the NKCC cotransporter in the ascending limb of the loop of Henle in the kidneys [75], was chosen as the structural framework to construct a chemical library of derivatives of it [76]. It has been demonstrated that AQP4 is inhibited by bumetanide and its products in Xenopus oocytes [76]. More study is necessary better to understand its potential applications in the management of gliomas.

The carbonic anhydrase inhibitor acetazolamide (AZA) inhibited either pure AQP4 reassembled in liposomes and ectopically produced AQP4 in Xenopus oocytes are inhibited by [77, 78]. More study is also necessary to understand its potential applications in the treatment of gliomas.

A total of 13 antiepileptic medications (AEDs) were assessed utilizing virtual docking procedures in silico [66, 67]. Seven of the choices inhibited AQP4 activity, and four substances, consisting of zonisamide (ZNS), lamotrigine (LTG), topiramate (TPM) and phenytoin (PHT), were then chosen for a dose-dependent investigation [66, 67]. Nevertheless, other investigators have failed to verify this function by utilizing primary glial cultures and thyroid epithelial cells transfected with AQP4 [79].

Propofol, also known as 2,6-diisopropyl phenol, is an intravenous anaesthetic frequently utilized in clinical settings. Previous studies have demonstrated that propofol can affect the apoptosis of neuronal cells [80] and decrease the expression of AQP as well as swelling of the brain in various models of animals [81]. Yang et al. found propofol reduced the expression of AQP4 in patients after gliomas removal [82]. Nevertheless, it is yet unknown how propofol works to inhibit AQP4.

Both the US Food and Drug Administration (FDA) and the UK's National Institute for Health and Care Excellence (NICE) have approved trifluoperazine (TFP) as an antipsychotic [83]. TFP was found to reduce cerebral edoema by blocking the subcellular relocalization of AQP4 to the plasma membrane of astrocytes, rather rendering the total suppression of AQP4 [83].

NF-κB inhibitors, like the curcumin and metformin, have also been found to effectively attenuate brain edoema in mice through inhibition of AQP4 pathway [84–86]. Therefore, they could also be considered a potential therapeutic drug for the treatment of glioma patients with brain edoema. Unfortunately, at the present time, there are no reports of clinical trials, thus well-designed clinical trials are needed to study and verify the neuroprotective effect of curcumin in glioma patients.

Intriguingly, temozolomide (TMZ), a standard first-line drug for malignant glioma, has also been found to have therapeutic potential for controlling proliferation, invasion of malignant glioma by inhibiting AQP4 expression through activation of p38 signal transduction pathway [12, 87].

Other compounds have also been explored to identify potential AQP4 inhibitors, but the results are controversial. By employing an automated fluorescent microplate reader-based test, calcein-loaded cells were used to screen 3575 substances, comprising 418 FDA-approved medicines [88]. Of them, NSC168597, NSC164914, NSC301460 and NSC670229 from the National Cancer Institute’s chemical library impact AQP4-mediated permeability to water [84]. However, subsequent stopped-flow scattering analyses revealed that none of these four compounds exhibited any inhibition of AQP4 [89].

## 7. Conclusion

It is evident that impairment of water homeostasis affects glioma advancement and AQP4 subcellular dislocation is one of the pathophysiological mechanisms driving glioblastoma. It’s still unclear, though, whether this shift results from the disease or is a cause of it. The particular roles of the M1 and M23 isoforms of AQP4 in glioma development have been identified. Additionally, it has been observed that a lack of a-syn directly impacts the polarization of AQP4. The activation of PKC can lead to post-translational modifications of AQP4, which in turn may affect the transportation of AQP4 during epileptogenesis. These results collectively indicate that AQP4 and/or its corresponding proteins could serve as potential targets for treatment. This can be achieved by either increasing the expression of perivascular AQP4 or by inhibiting its relocation [90]. As indicated before, numerous AQP4 modulators have been shown to have a significant inhibiting impact and potential therapeutic value for glioma; nonetheless, no one has received approval for usage in humans. More research is needed to understand the intriguing roles of AQP4 in GBM and its potential as a therapy.

## Data Availability

The data presented in this study are available upon request from the corresponding author.

## References

[1] Rahman R, Polley MC, Alder L, et al. Current drug development and trial designs in neuro-oncology: report from the first American Society of Clinical Oncology and Society for Neuro-Oncology Clinical Trials Conference. Lancet Oncol. 2023;24(4):e161–e171. doi: 10.1016/S1470-2045(23)00005-0. 36990614 PMC10401610

[2] Barden MM, Omuro AM. Top advances of the year: neuro-oncology. Cancer. 2023;129(10):1467–1472. doi: 10.1002/cncr.34711. 36825454

[3] Marvi MV, Neri I, Evangelisti C, et al. Phospholipases in gliomas: current knowledge and future perspectives from bench to bedside. Biomolecules. 2023;13(5):798. doi: 10.3390/biom13050798. 37238668 PMC10216162

[4] Qin X, Liu R, Akter F, et al. Peri-tumoral brain edema associated with glioblastoma correlates with tumor recurrence. J Cancer. 2021;12(7):2073–2082. doi: 10.7150/jca.53198. 33754006 PMC7974512

[5] Papadopoulos MC, Saadoun S, Davies DC, et al. Emerging molecular mechanisms of brain tumour ­oedema. Br J Neurosurg. 2001;15(2):101–108. doi: 10.1080/02688690120036775. 11360371

[6] Patabendige A, Chen RL. Astrocytic aquaporin 4 subcellular translocation as a therapeutic target for cytotoxic edema in ischemic stroke. Neural Regen Res. 2022;17(12):2666–2668. doi: 10.4103/1673-5374.339481. 35662202 PMC9165363

[7] Patabendige A, Singh A, Jenkins S, et al. Astrocyte activation in neurovascular damage and repair following ischaemic stroke. Int J Mol Sci. 2021;22(8):4280. doi: 10.3390/ijms22084280. 33924191 PMC8074612

[8] Varricchio A, Ramesh SA, Yool AJ. Novel ion channel targets and drug delivery tools for controlling glioblastoma cell invasiveness. Int J Mol Sci. 2021;22(21):11909. doi: 10.3390/ijms222111909. 34769339 PMC8584308

[9] Behnam M, Motamedzadeh A, Aalinezhad M, et al. The role of aquaporin 4 in brain tumors: implications for pathophysiology, diagnosis and therapy. Mol Biol Rep. 2022;49(11):10609–10615. doi: 10.1007/s11033-022-07656-y. 35715607

[10] Wang R, Peng L, Xiao Y, et al. Single-cell RNA sequencing reveals changes in glioma-associated macrophage polarization and cellular states of malignant gliomas with high AQP4 expression. Cancer Gene Ther. 2023;30(5):716–726. doi: 10.1038/s41417-022-00582-y. 36599974 PMC10191842

[11] Behling F, Barrantes-Freer A, Behnes CL, et al. Expression of Olig2, Nestin, NogoA and AQP4 have no impact on overall survival in IDH-wildtype glioblastoma. PLoS One. 2020;15(3):e0229274. doi: 10.1371/journal.pone.0229274. 32160197 PMC7065747

[12] Lan YL, Chen C, Wang X, et al. Gamabufotalin induces a negative feedback loop connecting ATP1A3 expression and the AQP4 pathway to promote temozolomide sensitivity in glioblastoma cells by targeting the amino acid Thr794. Cell Prolif. 2020;53(1):e12732. doi: 10.1111/cpr.12732. 31746080 PMC6985666

[13] Lan YL, Wang X, Lou JC, et al. The potential roles of aquaporin 4 in malignant gliomas. Oncotarget. 2017;8(19):32345–32355. doi: 10.18632/oncotarget.16017. 28423683 PMC5458289

[14] Lefranc F, Kiss R. The sodium pump alpha1 subunit as a potential target to combat apoptosis-resistant glioblastomas. Neoplasia. 2008;10(3):198–206. doi: 10.1593/neo.07928. 18323016 PMC2259449

[15] Lefranc F, Mijatovic T, Kondo Y, et al. Targeting the alpha 1 subunit of the sodium pump to combat glioblastoma cells. Neurosurgery. 2008;62(1):211–222. doi: 10.1227/01.NEU.0000311080.43024.0E. 18300910

[16] Peng Y, Wu W, Shang Z, et al. Inhibition of lncRNA LINC00461/miR-216a/aquaporin 4 pathway suppresses cell proliferation, migration, invasion, and chemoresistance in glioma. Open Life Sci. 2020;15(1):532–543. doi: 10.1515/biol-2020-0048. 33817241 PMC7874638

[17] Zou S, Lan YL, Ren T, et al. A bioinformatics analysis of the potential roles of aquaporin 4 in human brain tumors: an immune-related process. Front Pharmacol. 2021;12:692175. doi: 10.3389/fphar.2021.692175. 34113257 PMC8185330

[18] Valente O, Messina R, Ingravallo G, et al. Alteration of the translational readthrough isoform AQP4ex induces redistribution and downregulation of AQP4 in human glioblastoma. Cell Mol Life Sci. 2022;79(3):140. doi: 10.1007/s00018-021-04123-y. 35187599 PMC8858924

[19] Amiry-Moghaddam M. AQP4 and the fate of gliomas. Cancer Res. 2019;79(11):2810–2811. doi: 10.1158/0008-5472.CAN-19-1185. 31160309

[20] Warth A, Simon P, Capper D, et al. Expression pattern of the water channel aquaporin-4 in human gliomas is associated with blood-brain barrier disturbance but not with patient survival. J Neurosci Res. 2007;85(6):1336–1346. doi: 10.1002/jnr.21224. 17335082

[21] Fallier-Becker P, Nieser M, Wenzel U, et al. Is Upregulation of aquaporin 4-M1 isoform responsible for the loss of typical orthogonal arrays of particles in astrocytomas? Int J Mol Sci. 2016;17(8):1230. doi: 10.3390/ijms17081230. 27483250 PMC5000628

[22] Simone L, Pisani F, Mola MG, et al. AQP4 aggregation state is a determinant for glioma cell fate. Cancer Res. 2019;79(9):2182–2194. doi: 10.1158/0008-5472.CAN-18-2015. 30877104

[23] Xue Q, Cao L, Chen XY, et al. High expression of MMP9 in glioma affects cell proliferation and is associated with patient survival rates. Oncol Lett. 2017;13(3):1325–1330. doi: 10.3892/ol.2017.5567. 28454256 PMC5403257

[24] De Bellis M, Pisani F, Mola MG, et al. A novel human aquaporin-4 splice variant exhibits a dominant-negative activity: a new mechanism to regulate water permeability. Mol Biol Cell. 2014;25(4):470–480. doi: 10.1091/mbc.E13-06-0331. 24356448 PMC3923639

[25] Palazzo C, Buccoliero C, Mola MG, et al. AQP4ex is crucial for the anchoring of AQP4 at the astrocyte end-feet and for neuromyelitis optica antibody binding. Acta Neuropathol Commun. 2019;7(1):51. doi: 10.1186/s40478-019-0707-5. 30935410 PMC6444679

[26] Liotta LA, Stetler-Stevenson WG. Tumor invasion and metastasis: an imbalance of positive and negative regulation. Cancer Res. 1991;51(18 Suppl):5054s–5059s. 1884381

[27] Saadoun S, Papadopoulos MC, Hara-Chikuma M, et al. Impairment of angiogenesis and cell migration by targeted aquaporin-1 gene disruption. Nature. 2005;434(7034):786–792. doi: 10.1038/nature03460. 15815633

[28] Kong H, Fan Y, Xie J, et al. AQP4 knockout impairs proliferation, migration and neuronal differentiation of adult neural stem cells. J Cell Sci. 2008;121:24–36. 10.1242/jcs.035758 19033383

[29] Auguste KI, Jin S, Uchida K, et al. Greatly impaired migration of implanted aquaporin-4-deficient astroglial cells in mouse brain toward a site of injury. FASEB J. 2007;21(1):108–116. doi: 10.1096/fj.06-6848com. 17135365

[30] Small JV, Stradal T, Vignal E, et al. The lamellipodium: where motility begins. Trends Cell Biol. 2002;12(3):112–120. doi: 10.1016/s0962-8924(01)02237-1. 11859023

[31] Hara-Chikuma M, Verkman AS. Aquaporin-1 facilitates epithelial cell migration in kidney proximal tubule. J Am Soc Nephrol. 2006;17(1):39–45. doi: 10.1681/ASN.2005080846. 16319186

[32] Huttenlocher A. Cell polarization mechanisms during directed cell migration. Nat Cell Biol. 2005;7(4):336–337. doi: 10.1038/ncb0405-336. 15803131

[33] Nicchia GP, Srinivas M, Li W, et al. New possible roles for aquaporin-4 in astrocytes: cell cytoskeleton and functional relationship with connexin 43. Faseb J. 2005;19(12):1674–1676. doi: 10.1096/fj.04-3281fje. 16103109

[34] Warth A, Kröger S, Wolburg H. Redistribution of aquaporin-4 in human glioblastoma correlates with loss of agrin immunoreactivity from brain capillary basal laminae. Acta Neuropathol. 2004;107(4):311–318. doi: 10.1007/s00401-003-0812-0. 14735305

[35] Ding T, Gu F, Fu L, et al. Aquaporin-4 in glioma invasion and an analysis of molecular mechanisms. J Clin Neurosci. 2010;17(11):1359–1361. doi: 10.1016/j.jocn.2010.02.014. 20685122

[36] Polakis P. Wnt signaling and cancer. Genes Dev. 2000;14(15):1837–1851. doi: 10.1101/gad.14.15.1837. 10921899

[37] Felsberg J, Hentschel B, Kaulich K, et al. Epidermal growth factor receptor variant III (EGFRvIII) positivity in EGFR-amplified glioblastomas: prognostic role and comparison between primary and recurrent tumors. Clin Cancer Res. 2017;23(22):6846–6855. doi: 10.1158/1078-0432.CCR-17-0890. 28855349

[38] Ma Q, Ineichen BV, Detmar M, et al. Outflow of cerebrospinal fluid is predominantly through lymphatic vessels and is reduced in aged mice. Nat Commun. 2017;8(1):1434. doi: 10.1038/s41467-017-01484-6. 29127332 PMC5681558

[39] Wang S, Solenov EI, Yang B. Aquaporin inhibitors. Adv Exp Med Biol. 2023;1398:317–330. doi: 10.1007/978-981-19-7415-1_22. 36717504

[40] Wolburg H, Noell S, Fallier-Becker P, et al. The disturbed blood-brain barrier in human glioblastoma. Mol Aspects Med. 2012;33(5-6):579–589. doi: 10.1016/j.mam.2012.02.003. 22387049

[41] Noell S, Fallier-Becker P, Deutsch U, et al. Agrin defines polarized distribution of orthogonal arrays of particles in astrocytes. Cell Tissue Res. 2009;337(2):185–195. doi: 10.1007/s00441-009-0812-z. 19449033

[42] Rauch SM, Huen K, Miller MC, et al. Changes in brain β-amyloid deposition and aquaporin 4 levels in response to altered agrin expression in mice. J Neuropathol Exp Neurol. 2011;70(12):1124–1137. doi: 10.1097/NEN.0b013e31823b0b12. 22082664 PMC3223604

[43] Noell S, Mayer D, Strauss WS, et al. Selective enrichment of hypericin in malignant glioma: pioneering in vivo results. Int J Oncol. 2011;38(5):1343–1348. doi: 10.3892/ijo.2011.968. 21399870

[44] Furman CS, Gorelick-Feldman DA, Davidson KG, et al. Aquaporin-4 square array assembly: opposing actions of M1 and M23 isoforms. Proc Natl Acad Sci U S A. 2003;100(23):13609–13614. doi: 10.1073/pnas.2235843100. 14597700 PMC263861

[45] Smith AJ, Jin BJ, Ratelade J, et al. Aggregation state determines the localization and function of M1- and M23-aquaporin-4 in astrocytes. J Cell Biol. 2014;204(4):559–573. doi: 10.1083/jcb.201308118. 24515349 PMC3926963

[46] Crane JM, Van Hoek AN, Skach WR, et al. Aquaporin-4 dynamics in orthogonal arrays in live cells visualized by quantum dot single particle tracking. Mol Biol Cell. 2008;19(8):3369–3378. doi: 10.1091/mbc.e08-03-0322. 18495865 PMC2488293

[47] Saadoun S, Papadopoulos MC, Watanabe H, et al. Involvement of aquaporin-4 in astroglial cell migration and glial scar formation. J Cell Sci. 2005;118(Pt 24):5691–5698. doi: 10.1242/jcs.02680. 16303850

[48] Amiry-Moghaddam M, Xue R, Haug FM, et al. Alpha-syntrophin deletion removes the perivascular but not endothelial pool of aquaporin-4 at the blood-brain barrier and delays the development of brain edema in an experimental model of acute hyponatremia. Faseb J. 2004;18(3):542–544. doi: 10.1096/fj.03-0869fje. 14734638

[49] Neely JD, Amiry-Moghaddam M, Ottersen OP, et al. Syntrophin-dependent expression and localization of aquaporin-4 water channel protein. Proc Natl Acad Sci U S A. 2001;98(24):14108–14113. doi: 10.1073/pnas.241508198. 11717465 PMC61176

[50] Amiry-Moghaddam M, Otsuka T, Hurn PD, et al. An alpha-syntrophin-dependent pool of AQP4 in astroglial end-feet confers bidirectional water flow between blood and brain. Proc Natl Acad Sci U S A. 2003;100(4):2106–2111. doi: 10.1073/pnas.0437946100. 12578959 PMC149966

[51] Vandebroek A, Yasui M. Regulation of AQP4 in the central nervous system. Int J Mol Sci. 2020;21(5):1603. doi: 10.3390/ijms21051603. 32111087 PMC7084855

[52] Nesverova V, Törnroth-Horsefield S. Phosphorylation-dependent regulation of mammalian aquaporins. Cells. 2019;8(2):82. doi: 10.3390/cells8020082. 30678081 PMC6406877

[53] Han Z, Wax MB, Patil RV. Regulation of aquaporin-4 ­water channels by phorbol ester-dependent protein phosphorylation. J Biol Chem. 1998;273(11):6001–6004. doi: 10.1074/jbc.273.11.6001. 9497312

[54] Fazzina G, Amorini AM, Marmarou CR, et al. The protein kinase C activator phorbol myristate acetate decreases brain edema by aquaporin 4 downregulation after middle cerebral artery occlusion in the rat. J Neurotrauma. 2010;27(2):453–461. doi: 10.1089/neu.2008.0782. 19831719 PMC2864458

[55] Moeller HB, Fenton RA, Zeuthen T, et al. Vasopressin-dependent short-term regulation of ­aquaporin 4 ­expressed in Xenopus oocytes. Neuroscience. 2009;164(4):1674–1684. doi: 10.1016/j.neuroscience.2009.09.072. 19800950

[56] Niermann H, Amiry-Moghaddam M, Holthoff K, et al. A novel role of vasopressin in the brain: modulation of activity-dependent water flux in the neocortex. J Neurosci. 2001;21(9):3045–3051. doi: 10.1523/JNEUROSCI.21-09-03045.2001. 11312289 PMC6762582

[57] Zhou W, Yu X, Sun S, et al. Increased expression of MMP-2 and MMP-9 indicates poor prognosis in glioma recurrence. Biomed Pharmacother. 2019;118:109369. doi: 10.1016/j.biopha.2019.109369. 31545229

[58] Zhao WJ, Zhang W, Li GL, et al. Differential expression of MMP-9 and AQP4 in human glioma samples. Folia Neuropathol. 2012;50(2):176–186. 22773464

[59] Salman MM, Kitchen P, Halsey A, et al. Emerging roles for dynamic aquaporin-4 subcellular relocalization in CNS water homeostasis. Brain. 2022;145(1):64–75. doi: 10.1093/brain/awab311. 34499128 PMC9088512

[60] Morikawa Y, Heallen T, Leach J, et al. Dystrophin-glycoprotein complex sequesters Yap to inhibit cardiomyocyte proliferation. Nature. 20173;547(7662):227–231. doi: 10.1038/nature22979. 28581498 PMC5528853

[61] Michaluk P, Kolodziej L, Mioduszewska B, et al. Beta-dystroglycan as a target for MMP-9, in response to enhanced neuronal activity. J Biol Chem. 2007;282(22):16036–16041. doi: 10.1074/jbc.M700641200. 17426029

[62] Montagne A, Nation DA, Sagare AP, et al. APOE4 leads to blood-brain barrier dysfunction predicting cognitive decline. Nature. 2020;581(7806):71–76. doi: 10.1038/s41586-020-2247-3. 32376954 PMC7250000

[63] Zhu B, Cao A, Chen C, et al. MMP-9 inhibition alleviates postoperative cognitive dysfunction by improving glymphatic function via regulating AQP4 polarity. Int Immunopharmacol. 2024;126:111215. doi: 10.1016/j.intimp.2023.111215. 38000234

[64] Lan YL, Wang H, Chen A, et al. Update on the current knowledge of lymphatic drainage system and its emerging roles in glioma management. Immunology. 2023;168(2):233–247. doi: 10.1111/imm.13517. 35719015

[65] Tyurina YY, Polimova AM, Maciel E, et al. LC/MS analysis of cardiolipins in substantia nigra and plasma of rotenone-treated rats: implication for mitochondrial dysfunction in Parkinson’s disease. Free Radic Res. 2015;49(5):681–691. doi: 10.3109/10715762.2015.1005085. 25740198 PMC4430340

[66] Huber VJ, Tsujita M, Kwee IL, et al. Inhibition of aquaporin 4 by antiepileptic drugs. Bioorg Med Chem. 2009;17(1):418–424. doi: 10.1016/j.bmc.2007.12.038. 18178093

[67] Huber VJ, Tsujita M, Nakada T. Identification of aquaporin 4 inhibitors using in vitro and in silico methods. Bioorg Med Chem. 2009;17(1):411–417. doi: 10.1016/j.bmc.2007.12.040. 18182301

[68] Igarashi H, Huber VJ, Tsujita M, et al. Pretreatment with a novel aquaorin-4 inhibitor, TGN-20, significantly reduces ischemic cerebral edema. Neurol Sci. 2011;32(1):113–116. doi: 10.1007/s10072-010-0431-1. 20924629 PMC3026762

[69] Zelenina M. Regulation of brain aquaporins. Neurochem Int. 2010;57(4):468–488. doi: 10.1016/j.neuint.2010.03.022. 20380861

[70] Zhang L, Yu Z, Wang Y, et al. Quantitative proteomics reveals molecular mechanism of gamabufotalin and its potential inhibition on Hsp90 in lung cancer. Oncotarget. 2016;7(47):76551–76564. doi: 10.18632/oncotarget.10388. 27384878 PMC5363529

[71] Yu Z, Li T, Wang C, et al. Gamabufotalin triggers c-Myc degradation via induction of WWP2 in multiple myeloma cells. Oncotarget. 2016;7(13):15725–15737. doi: 10.18632/oncotarget.7398. 26894970 PMC4941272

[72] Detmers FJ, de Groot BL, Müller EM, et al. Quaternary ­ammonium compounds as water channel blockers. Specificity, potency, and site of action. J Biol Chem. 2006;281(20):14207–14214. doi: 10.1074/jbc.M513072200. 16551622

[73] Küppers E, Gleiser C, Brito V, et al. AQP4 expression in striatal primary cultures is regulated by dopamine-implications for proliferation of astrocytes. Eur J Neurosci. 2008;28(11):2173–2182. doi: 10.1111/j.1460-9568.2008.06531.x. 19046364

[74] Søgaard R, Zeuthen T. Test of blockers of AQP1 water permeability by a high-resolution method: no effects of tetraethylammonium ions or acetazolamide. Pflugers Arch. 2008;456(2):285–292. doi: 10.1007/s00424-007-0392-2. 18043939

[75] Haas M, McManus TJ. Bumetanide inhibits Na+-K+-2Cl–co-transport at a chloride site. Am J Physiol. 1983;245(3):C235–C240. doi: 10.1152/ajpcell.1983.245.3.C235. 6614157

[76] Migliati E, Meurice N, DuBois P, et al. Inhibition of aquaporin-1 and aquaporin-4 water permeability by a derivative of the loop diuretic bumetanide acting at a internal pore-occluding binding site. Mol Pharmacol. 2009;76(1):105–112. doi: 10.1124/mol.108.053744. 19403703 PMC2701455

[77] Huber VJ, Tsujita M, Yamazaki M, et al. Identification of arylsulfonamides as aquaporin 4 inhibitors. Bioorg Med Chem Lett. 2007;17(5):1270–1273. doi: 10.1016/j.bmcl.2006.12.010. 17178220

[78] Tanimura Y, Hiroaki Y, Fujiyoshi Y. Acetazolamide reversibly inhibits water conduction by aquaporin-4. J Struct Biol. 2009;166(1):16–21. doi: 10.1016/j.jsb.2008.11.010. 19114109

[79] Yang B, Zhang H, Verkman AS. Lack of aquaporin-4 ­water transport inhibition by antiepileptics and arylsulfonamides. Bioorg Med Chem. 2008;16(15):7489–7493. doi: 10.1016/j.bmc.2008.06.005. 18572411 PMC3325054

[80] Xi HJ, Zhang TH, Tao T, et al. Propofol improved neurobehavioral outcome of cerebral ischemia-reperfusion rats by regulating Bcl-2 and Bax expression. Brain Res. 2011;1410:24–32. doi: 10.1016/j.brainres.2011.06.060. 21783180

[81] Lee JH, Cui HS, Shin SK, et al. Effect of propofol post-treatment on blood-brain barrier integrity and cerebral edema after transient cerebral ischemia in rats. Neurochem Res. 2013;38(11):2276–2286. doi: 10.1007/s11064-013-1136-7. 23990224

[82] Yang WC, Zhou LJ, Zhang R, et al. Effects of propofol and sevoflurane on aquaporin-4 and aquaporin-9 ­expression in patients performed gliomas resection. Brain Res. 2015;1622:1–6. doi: 10.1016/j.brainres.2015.05.042. 26100336

[83] Kitchen P, Salman MM, Halsey AM, et al. Targeting aquaporin-4 subcellular localization to treat central nervous system edema. Cell. 2020;181(4):784–799.e19. doi: 10.1016/j.cell.2020.03.037. 32413299 PMC7242911

[84] Wang BF, Cui ZW, Zhong ZH, et al. Curcumin attenuates brain edema in mice with intracerebral hemorrhage through inhibition of AQP4 and AQP9 expression. Acta Pharmacol Sin. 2015;36(8):939–948. doi: 10.1038/aps.2015.47. 26119880 PMC4564884

[85] Yu LS, Fan YY, Ye G, et al. Curcumin alleviates brain edema by lowering AQP4 expression levels in a rat model of hypoxia-hypercapnia-induced brain damage. Exp Ther Med. 2016;11(3):709–716. doi: 10.3892/etm.2016.3022. 26997983 PMC4774356

[86] Zhao B, Wang X, Zheng J, et al. Effects of metformin treatment on glioma-induced brain edema. Am J Transl Res. 2016;8(8):3351–3363. 27648126 PMC5009388

[87] Chen Y, Gao F, Jiang R, et al. Down-regulation of AQP4 expression via p38 MAPK signaling in temozolomide-induced glioma cells growth inhibition and invasion impairment. J Cell Biochem. 2017;118(12):4905–4913. doi: 10.1002/jcb.26176. 28569417

[88] Mola MG, Nicchia GP, Svelto M, et al. Automated cell-based assay for screening of aquaporin inhibitors. Anal Chem. 2009;81(19):8219–8229. doi: 10.1021/ac901526k. 19705854 PMC2850055

[89] Esteva-Font C, Jin BJ, Lee S, et al. Experimental evaluation of proposed small-molecule inhibitors of water channel aquaporin-1. Mol Pharmacol. 2016;89(6):686–693. doi: 10.1124/mol.116.103929. 26993802 PMC4885500

[90] Bhattacharjee A, Jana A, Bhattacharjee S, et al. The role of aquaporins in tumorigenesis: implications for therapeutic development. Cell Commun Signal. 2024;22(1):106. doi: 10.1186/s12964-023-01459-9. 38336645 PMC10854195

